# Design and Analysis of a Three-Dimensional Spindle-Shaped Receiving Coil for a Targeted Therapy Capsule Robot in the Intestine

**DOI:** 10.3390/mi13111884

**Published:** 2022-11-01

**Authors:** Ding Han, Guozheng Yan, Kai Zhao, Zhiwu Wang, Pingping Jiang, Lin Yan

**Affiliations:** 1 School of Electronic Information and Electrical Engineering, Shanghai Jiao Tong University, Shanghai 200240, China; 2 Shanghai Engineering Research Center of Intelligent Addiction Treatment and Rehabilitation, Shanghai 200240, China; 3 Scientific Computing & Imaging Institute, University of Utah, Salt Lake City, UT 84112, USA

**Keywords:** receiving coil, targeted therapy capsule robot, wireless power transfer, intestinal diseases detection

## Abstract

Capsule robots capable of taking wireless power-transfer systems for diagnosis in the intestine enable the ability to avoid invasive detection, which causes damage to tissue. A targeted therapy capsule robot based on a wireless power-transfer system could move actively in the intestine, implementing diseases detection and drug delivery. Compared with traditional telescope, the capsule robot explores without pain to patients. However, the insufficient power supply has become a big issue for a targeted therapy capsule robot. To address this problem, we proposed a new type of three-dimensional spindle-shaped receiving coil that can couple well with unidirectional magnetic fields and supply sufficient energy even when there is a misalignment in position and angle, owing to which the electromagnetic energy decays quickly. The proposed receiving coil could be embedded on the capsule robot, suitable for the capsule size *Φ*15 mm × 25 mm. To obtain the maximum energy in three-dimensional space, an optimization model was built. The parameters of the receiving coil were optimized and analyzed. Then, the designed receiving coil was verified with an energy-transfer stability analysis based on both attitude angle and position in a bench test. Furthermore, a curved pipe experiment was conducted using a capsule robot prototype with the proposed three-dimensional spindle-shaped receiving coil. The results demonstrated that stable and sufficient power could be supplied by the proposed receiving coil for the capsule robot at any position and any attitude angle between transmitting and receiving coils.

## 1. Introduction

The incidence of intestinal malignant tumors and intestinal functional diseases has been increasing recently. Digestive tract problems have become critical aspects that threaten people’s health and life [1,2]. The treatment methods for intestinal diseases have been focused on by researchers. Endoscopes are common devices in the clinic. However, they usually cause pain and complications during the invasive process in therapy [3,4,5]. Capsule robots capable of taking wireless power-transfer systems for diagnosis in the intestine enable the ability to avoid invasive detection, which causes damage to tissue [6,7]. A targeted therapy capsule robot should move actively in the intestine, implementing disease detection and drug delivery. Therefore, sufficient power-supplied approaches are in urgent demand in such systems.

Power supply is always a challenge in targeted therapy capsule robots due to dimension constraints and high-consuming modules, such as motion mechanisms [8,9]. Instead of a wired power supply, silver oxide button batteries are of interest to researchers [10,11]. In refs. [12,13], considering the power output from the battery is too weak to drive capsule robots based on smart actuators, the researchers proposed a proper motor combined with a screw thread to integrate in the capsule robot to reduce power consumption. Unfortunately, they found that it is still not possible to realize a wireless capsule robot that can travel long distances (from the esophagus to the anus) through experiments. Thus, a new way to supply power is needed. The work of refs. [14,15] designed another way to supply power by using permanent magnets. A magnetized ring magnet was imbedded in the robot, and then the robot could be steered to move by applying a magnetic field via an external permanent magnet. However, the magnetic power is weak and the movement is not smooth. Currently, this kind of power supply method only makes it possible to move in a space full of fluid, such as the stomach. The intestine is still the bottleneck. In addition, we note that it still needs battery power to realize image capture and other functions. 

To solve these issues, wireless power-transfer systems, which provide unique features, have been greatly involved in medical applications. In general, wireless power-transfer systems require proper alignment between the transmitting coil outside body and a receiving coil implanted in the body to receive sufficient power to operate the capsule robot. As reviewed in ref. [16], one-dimensional transmitting coil and one-dimensional receiving coil could couple well only at specific point. It is difficult to guarantee uniform and sufficient magnetic field strength in the desired area. In order to enable the receiving coil, whose position and angle are always changing to receive as much energy as possible, a three-dimensional transmitting coil can be used. Under this condition, it is necessary to adjust the magnetic field direction of the transmitting coil in real time according to the current angle information of the receiving coil to achieve better coupling between the two coils [17,18]. It is difficult to obtain the real-time position and angle information of the receiving coil. Moreover, the angle of the three-dimensional transmitting coil corresponding to the receiving coil is hard to control. Even if these operations are possible, the extremely complex system structure is prone to cause electromagnetic interference and information errors [19,20].

To avoid these disadvantages, the combination of a three-dimensional receiving coil and one-dimensional transmitting coil is a more feasible solution without the knowledge of real-time angle information of the receiving coil [21,22]. However, it also has some technical difficulties, such as limited space for the receiving coil and weak coupling due to misalignment in position and angle between two coils when the capsule robot moves. Although the literature illustrated the three-dimensional receiving coil, it only analyzed the perfect coupling situation in the ideal position.

The challenges faced still exist with the three-dimensional receiving coil. In this article, we proposed a novel type of three-dimensional spindle-shaped receiving coil for a targeted therapy capsule robot in the intestine. The proposed receiving coil has a hollow structure that is suitable for capsule-sized robots. Moreover, it could couple well with unidirectional magnetic fields and supply sufficient energy even there is a misalignment in position and angle between the transmitting and receiving coils.

This paper is organized as follows. The overview of the capsule robot intestinal diagnosis system with receiving coil is introduced in Section 2. Section 3 focuses on details about developing a mathematical model to analyze and optimize the design parameters of the three-dimensional spindle-shaped receiving coil to achieve maximum power. In Section 4, the theory model is evaluated by experiments using a receiving coil prototype and a capsule robot. Finally, conclusions are given in Section 5.

## 2. Targeted Therapy Capsule Robot System Review

Instead of a traditional endoscope, targeted therapy capsule robots with wireless power-transfer systems could explore the intestine for disease detection noninvasively. Capsule robots move in the intestine and actively implement the targeted therapy with an image acquisition module and drug delivery. All energy is supplied by a wireless power-transfer system.

**Figure 1:**
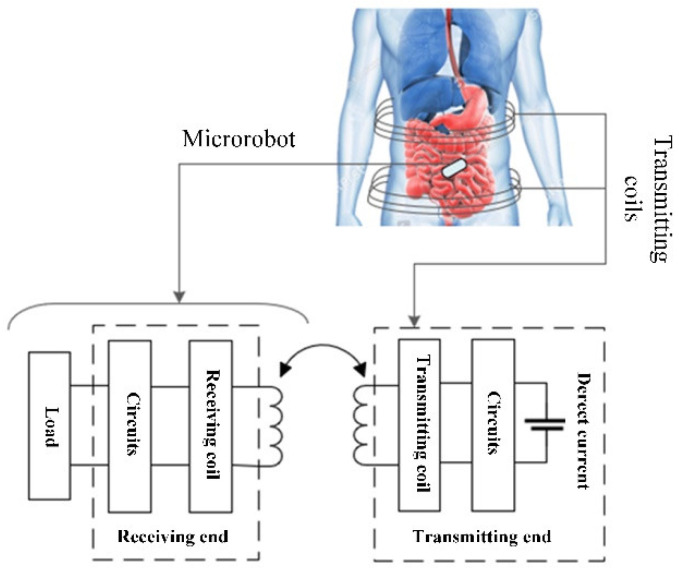
Block diagram of the wireless power-transfer system.

As is shown in Figure 1, a receiving coil is imbedded in the robot, coupling with an external transmitting coil to obtain energy for the detection system. At the transmitting end, the drive circuit generates a square wave signal with a fixed frequency. The direct current is converted into alternating current after a full-bridge inverter circuit. Then an alternating magnetic field in the resonance state is generated using the tuning circuit. At the receiving end, the receiving coil obtains the induced electromotive force. A stable voltage for the capsule robot system is output after processed by the energy receiving circuits.

## 3. 3D Spindle-Shaped Receiving Coil

In the traditional structure design of capsule robots, it is necessary to reserve a separate space at its front or rear to place the receiving coil, which inevitably increases the overall length of the robot. This design violates the design philosophy that the smaller the robot implanted in the body, the better it is. In order to save space, the receiving coil used in this paper needs to be wound on the surface of the hollow cylindrical magnetic core. Thus, the robot mechanism can be placed inside the hollow part. A novel 3D spindle-shaped structure receiving coil is proposed, which can receive electromagnetic energy from any direction. 

**Figure 2:**
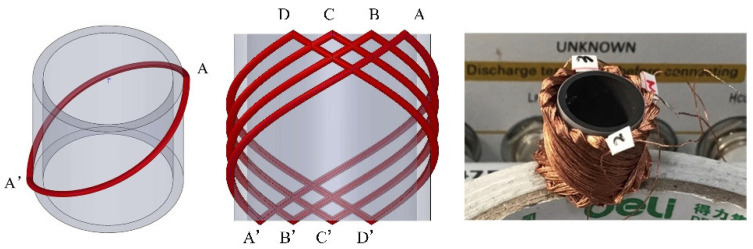
Illustration of the spindle-shaped receiving coil winding process.

The spindle-type receiving coil winding process is shown in Figure 2. A cylindrical ferrite core was used here to enhance the coupling coefficient [23]. As shown in the leftmost figure, the wire was wound to start from point A on the cylindrical ferrite core, along the red arc from start point A to end point $A^{\prime }$ (homologous to point A by means of a central symmetry). Then, we returned to point A with reversed orientation, forming a closed loop. We rotated the elliptical coil $AA^{\prime }$ 30° counterclockwise around the central axis (*z*) of the cylinder to obtain coil $BB^{\prime }$. Coils $CC^{\prime }$ and $DD^{\prime }$ could be obtained in the same way. The four coils were connected end to end in series to obtain the spindle-shaped receiving coil group I, as shown in the middle figure. Similarly, repeating the upfront winding way, the coil group I was rotated 120° counterclockwise twice, and then the coil group II and the coil group III could be obtained. Three groups of coils were distributed uniformly and connected in parallel. The prototype of the receiving coil is presented in the rightmost figure. The size of the receiving coil is Φ14.9 mm×10.9 mm.

In the patient’s body, with the continuous movement of the capsule robot, its position and posture are also constantly changing. Therefore, the stability of the energy transmitted by the WPT system plays a key role in design process. Our working hypotheses are as follows:

A. During the movement, the capsule robot travels slowly, so the relative motion between the transmitting coil and the receiving coil can be ignored, and then the dynamic electromotive force between these two can be ignored. Only induced electromotive force is considered.

B. Since there is a significant difference in size between the transmitting coil and the receiving coil (the size of the transmitting coil is much larger than the size of the receiving coil), it can be assumed that the distribution of the magnetic field inside the receiving coil is uniform, and the magnetic induction intensity is equal everywhere. Only when the receiving coil changes position does the magnetic field strength change with it.

### 3.1. Induced Electromotive Force Computation

**Figure 3:**
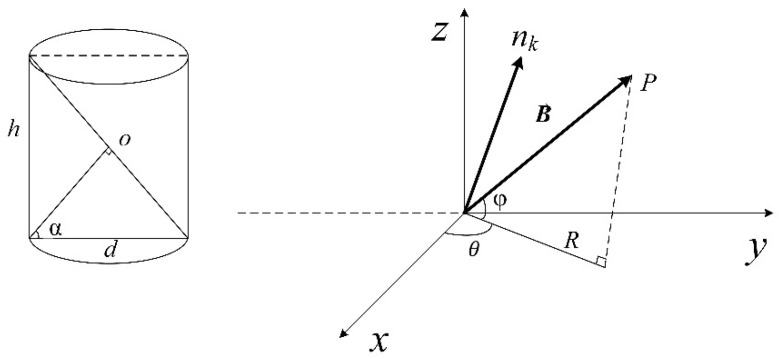
The normal vector and the magnetic field strength density of the receiving coil.

The single-turn receiving coil was analyzed and the induced electromotive force generated by the electromagnetic induction was calculated. The receiving coil was wound on a hollow cylinder with height h and diameter d, see left figure in Figure 3. As discussed above, the receiving coil is composed of N groups of coils. Then, we defined the number of winding layer in each group as L, and the turn number of each layer as T. Due to distributed uniformly, the angle of the arc space occupied by each single-turn coil in each group is $\theta_{0}=\frac{2\pi }{N\cdot T}$. The angle between the normal vector of any single-turn coil and the cross section is *α*. We set o as the center of the receiving coil, creating a Cartesian coordinate system with o as the origin, see right figure in Figure 3. o is the center of the receiving coil, with distance r to the transmitting coil $C_{t}$. $\vec{oP}$ is the direction of the magnetic field. θ is the angle between the projection of $\vec{oP}$ on the xoy plane and the *x*-axis, and φ is the angle between $\vec{oP}$ and the xoy plane (angle between coil planes). 

The induced electromotive force of a single-turn coil can be calculated as

(1) $$\begin{array}{c} e_{k}=\frac{\partial B_{o}\cdot S}{\partial t}=\frac{\partial B_{o}\cdot n_{k}\left|S\right|}{\partial t}\; \end{array}$$

where, $B_{o}$ represents the magnetic induction at point o. $S=\pi \cdot \frac{d/2}{sin\alpha }\cdot \frac{d}{2}$ is the single-turn coil ellipse area. $n_{k}$ is the normal vector of the kth-turn coil, as shown in the right figure of Figure 3.

Let $N_{P}$ be the positional parameter, which is only related to the coordinates of the center of the coil. $\sigma_{k}$ is the angle parameter.

(2) $$\begin{array}{c} N_{P}=\frac{\mu_{0}}{4\pi }\left|\oint_{C_{t}}^{\;}\frac{dl\times r}{r^{3}}\right| \end{array}$$

(3) $$\sigma_{k}\left(\alpha ,\theta_{0};\theta ,\varphi \right)=cos\varphi cos\alpha \left(cosk\theta_{0}cos\theta +sink\theta_{0}sin\theta +tan\varphi tan\alpha \right)\;$$

where, $\mu_{0}$ is the permeability of vacuum.

Then, Equation (1) can be rewritten as,

(4) $$e_{k}=\frac{dI}{dt}SN_{P}\sigma_{k}\left(\alpha ,\theta_{0};\theta ,\varphi \right)$$

where, I is the current.

The induced electromotive force $e^{\left(i\right)}$ (i = 1,2,3) of each group of coils is the sum of each single turn.

(5) $$\begin{array}{c} e^{\left(i\right)}=L_{0}\overset{N_{0}}{\underset{k=1}{\sum }}e_{k}=\frac{dI}{dt}SN_{P}L_{0}\overset{N_{0}}{\underset{k=1}{\sum }}\sigma_{k}\left(\alpha ,\theta_{0};\theta ,\varphi \right) \end{array}$$

The total induced electromotive force of the spindle-shaped receiving coil as the three groups of coils connected in series is as follows:

(6) $$\begin{array}{c} e=e^{\left(1\right)}+e^{\left(2\right)}+e^{\left(3\right)} \end{array}$$

Analogously, the total induced electromotive force as the three groups of coils connect in parallel is as follows:

(7) $$\begin{array}{c} e=\max\left(e^{\left(1\right)},e^{\left(2\right)},e^{\left(3\right)}\right) \end{array}$$

According to the above formula, the induced electromotive force of the receiving coil at the position P with an offset angle of $\left(\theta ,\varphi \right)$ can be obtained.

In addition, considering the large internal resistance and high energy consumption of the rectifier circuit when the three groups of coils are connected in series, the parallel connection style was adopted. Once the posture (i.e., positions and angles) of the receiving coil is known, the induced electromotive force of the receiving coil is obtained.

### 3.2. Optimization of Proposed Receiving Coil

In this section, parameters of proposed receiving coil are optimized to obtain sufficient power. The induced electromotive force $e^{\left(i\right)}$ in Equation (5) should be considered as the optimization objective. Find the optimal parameters in their domains, such as number of groups N, number of layers L, and number of turns T, to obtain

(8) $$\max\left\{e^{\left(i\right)}=\frac{dI}{dt}S_{0}N_{C}L_{0}\sum_{k=1}^{N_{0}}\sigma_{k}\left(\alpha ,\theta_{0};\theta ,\varphi \right)\right\}$$

For limited space, note that the outer diameter $D_{o}$ of the receiving coil should be controlled no more than 15 mm due to capsule robot’s smooth motion in the intestine for detection. The inter diameter $D_{i}$ of the receiving coil should be more than 10 mm to free up internal space for capsule robot mechanisms. Then for any parameters (i.e., N, L and T), it holds that

(9) $$D_{i}=\frac{N\cdot T\cdot d_{0}}{\pi }\geq 10\times 10^{-3}$$

(10) $$D_{o}=\frac{N\cdot T\cdot d_{0}}{\pi }+2\cdot d_{0}\left[\frac{\sqrt{3}}{2}\left(L-1\right)+1\right]\leq 15\times 10^{-3}\;$$

where, $d_{0}$ is the wire diameter (Litz wire with 18 strands and 0.05 mm dia. each strand).

Moreover, the received power P must be greater than or equal to the minimum power 580 mW needed by the capsule robot.

(11) $$P=\frac{e^{\left(i\right)}{}^{2}}{R}\geq 580\times 10^{-3}$$

Thus, the optimization model was built with the optimization objective Equation (8) and constrains Equations (9) to (11).

**Figure 4:**
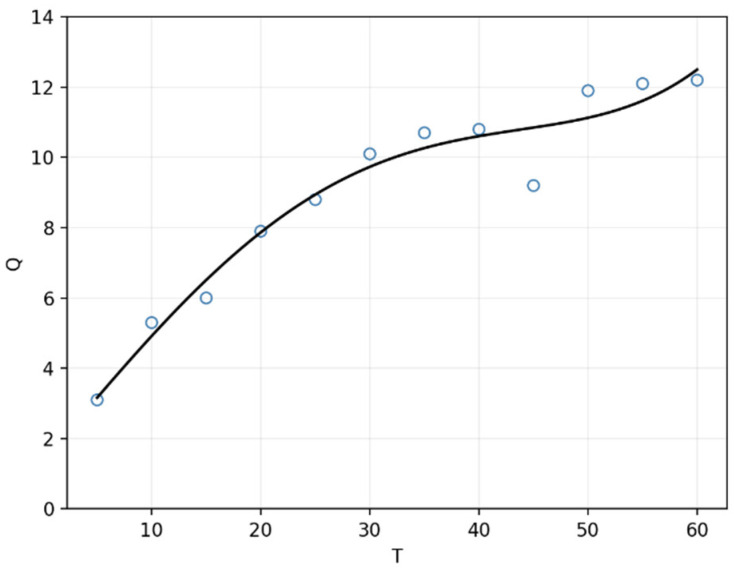
The relationship between the number of coil turns T and related parameters Q.

In addition, the quality factor Q of the receiving coil is a key factor to characterize its energy-receiving ability. A series of tests with receiving coils with different turns were conducted to test their quality factor. The results are shown in Figure 4. Note that the blue circles are recorded experimental data points and the black curves are polynomial fitted functions. It is easy to find that the quality factor is positively related to the number of turns. That is to say, the greater number of turns, the better the coil within a certain range. 

From the discussion above, the optimization parameters were obtained, and are listed in Table 1. Therefore, the proposed coil has three groups with 51 turns in each group.

## 4. Experiments

**Figure 5:**
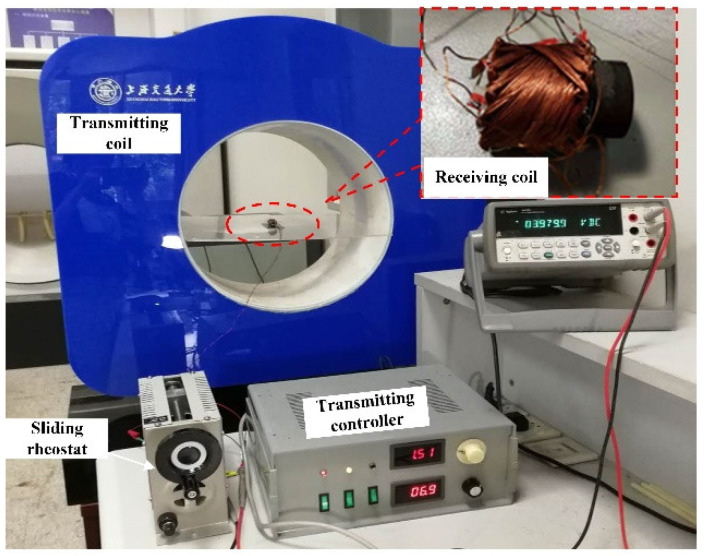
Experimental setup for measuring transmission efficiency and transmission power of the receiving coil.

The proposed 3D spindle-shaped receiving coil was constructed from Litz wire with a ferrite magnetic core. The experimental setup was built as shown in Figure 5. The proposed receiving coil was placed in the center of the Helmholtz coil, which was adopted as the transmitting coil. By using a rotatable mechanism, the receiving coil could rotate with angles. A sliding rheostat set 30 Ω was used as equivalent resistance of the capsule robot. Table 2 lists the parameters of the transmitting coil.

### 4.1. Energy Transfer Stability Analysis Based on Attitude

**Figure 6:**
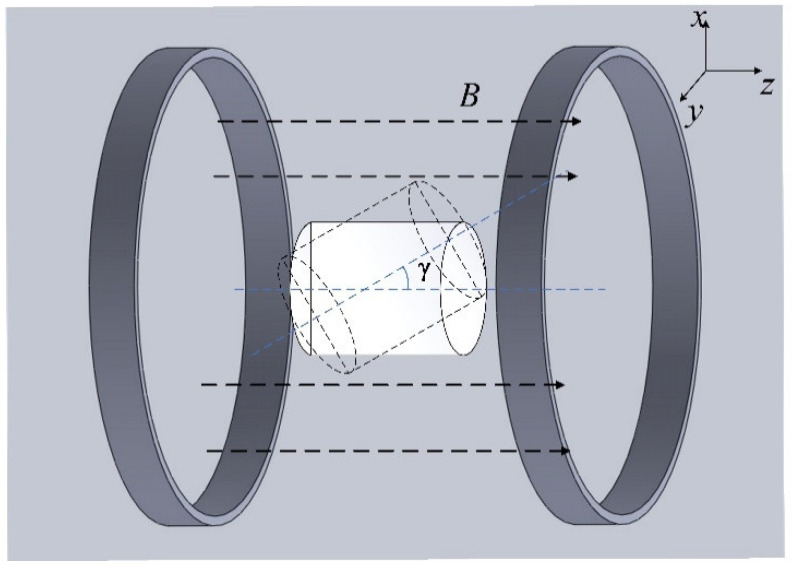
Schematic diagram of the attitude change of the receiving coil.

In order to verify the energy transfer stability based on attitude angle, the relative angle of the mutual inductance planes of the transmitting and receiving coils was changed to obtain the power supplied by the system at different attitudes. According to structure symmetry, the effect of rotating the coil around the *x*-axis is the same as the effect of rotating around the *y*-axis. Thus, the receiving coil was rotated about the *y*-axis with angle γ, as shown in Figure 6.

**Figure 7:**
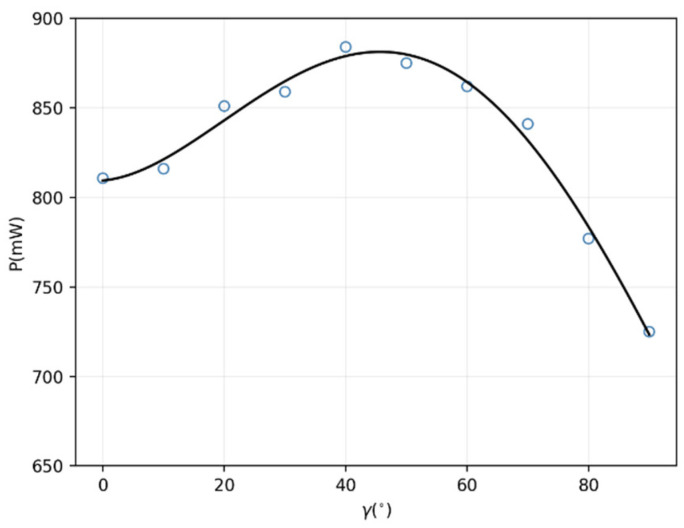
The change of received power when the receiving coil rotates around the *y*-axis.

Note that the attitude angle had periodic changes during the 360° rotation. The γ taking values from 0 to 90° could reflect the whole phenomena. In the test, every 10° was sampled at the range of 0 to 90°. The results are described in Figure 7.

It can be seen from Figure 7 that with increasing attitude angle, the power the receiving coil obtained increased gradually first, and then dropped down to 725 mW (transmission efficiency 5.5%, which is sufficient for a targeted therapy capsule robot (580 mW needed) in the intestine. The peak of power 885 mW (transmission efficiency 6.7%) occurred at 45°, which indicated that the effective projected area in the direction of the magnetic field was the largest at this time. The results demonstrated that, in a unidirectional magnetic field, using the proposed three-dimensional spindle-shaped receiving coil, the power could be obtained at any attitude at a high level.

### 4.2. Energy Transfer Stability Analysis Based on Position

The coordinate axis distribution is consistent with the previous theoretical analysis, so that the central axis of the cylinder of the spindle-shaped receiving coil coincides with the *z*-axis direction of the transmitting coil. The origin is defined at the geometric center of the transmitter coil.

**Figure 8:**
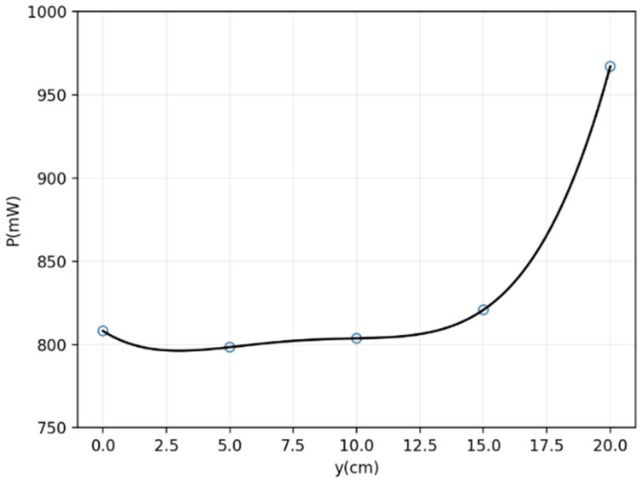
Standard deviation of the sampled data on different planes.

Sampling was taken uniformly at 5 cm intervals inside the transmitter coil. Considering the uniformity and symmetry of the magnetic field distribution, the position movement in the *y*-axis direction only needs to consider the positive direction. We set y at 0 cm, 5 cm, 10 cm, 15 cm, and 20 cm respectively, obtaining five planes parallel to the x–o–z plane. Similarly, we conducted the same sampling along the *x*-axis and the *z*-axis on each plane. The receiving power of the designed coil at every location in the three-dimensional space can be measured. For the convenience of observation and analysis, the standard deviation of the received power on each plane was calculated, and is presented in Figure 8.

As can be seen from Figure 8, the variation was moderate in the range of 0 to 12 cm, which indicates that the uniformity of the magnetic field at the center of the system is the best. However, the magnetic field strength at the center is slightly weaker than that near the transmitting coil’s edge. Obviously, it can obtain a relatively large magnetic field strength but with poor uniformity near the edge of the coil.

**Figure 9:**
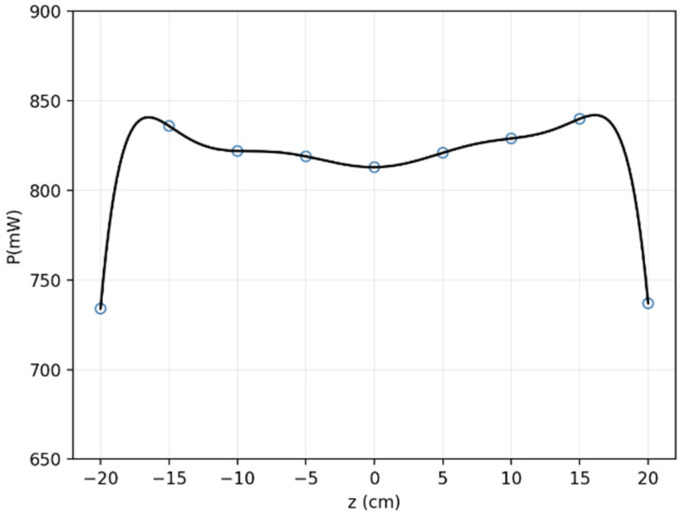
Magnetic field distribution on the *z*-axis.

Further, we analyzed the sampling data at the center (y = 0) of the system (i.e., on plane x–o–z along with the *z*-axis). Results are depicted in Figure 9. It shows a large area of uniform magnetic field (from −12 cm to 12 cm), though there are some unavoidable measurement errors, which is reasonable. The above discussion results in Figure 8 are well verified here. We also note that the minimum received power measured in the experiment when changing location of the receiving coil was 734 mW, which can meet the energy requirement of the capsule robot system. In fact, it is impossible to reach this extreme position.

### 4.3. Curved Pipe Experiments

**Figure 10:**
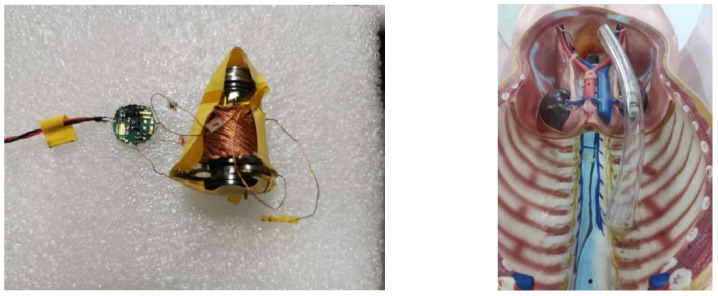
The capsule robot prototype with the proposed receiving coil and curved pipe experiments.

A targeted therapy capsule robot in the intestine generally consists of a drive mechanism, control module, image acquisition module, drug delivery module, and wireless power-transfer system. In this experiment, the three-dimensional spindle-shaped receiving coil is embedded in the capsule robot to supply energy for the other four modules. The left figure in Figure 10 presents the capsule robot prototype with the proposed receiving coil (unpackaged). The capsule robot moves through axial mechanism telescoping and radial mechanism expansion [24]. When mechanisms closed, the capsule robot has the capsule-like size Φ15 mm×50 mm with 20 g.

A piece of transparent, curved tube to mimic the intestine was placed in a magnetic field produced by the Helmholtz coil, shown in the right figure of Figure 10. The targeted therapy capsule robot equipped with proposed receiving coil (encapsulated) is used to verify the stability and effectiveness of the designed receiving coil. The current in transmitting coil was set to 1.2 A, and frequency was set to 220 kHz. Under the wireless power-transfer system, the capsule robot could move along the tube steadily, and even make turns. The average speed of the capsule robot is 1.43 mm/s. This experiment demonstrates that the proposed receiving coil could couple well with unidirectional magnetic fields and supply sufficient energy at any location and any attitude angle in the transmitting coil.

## 5. Conclusions

Wireless power-transfer systems are used in the targeted therapy capsule robots to realize disease detection and drug delivery noninvasively. As is known, since the electromagnetic energy decays quickly with distance, the misalignment in position and angle plays a key role in inductive coupling. We explored a novel type of three-dimensional spindle-shaped receiving coil. Through parameter optimization, the receiving coil can couple well with unidirectional magnetic fields and supply sufficient energy at any position and any attitude angle. The hollow structure of the proposed receiving coil has the size Φ15 mm×25 mm, which could make the capsule robot keep in a capsule size after embedded on it. A bench test verifying the energy transfer stability based on both attitude angle and position was conducted. The results presented that the power could reach 885 mW with transmission efficiency 6.7% in the designed wireless power-transfer system, better than 845 mW in reference [16]. Even the minimum received power 725 mW is more than demanded power 580 mW for an intestinal disease detection capsule robot. Furthermore, a curved pipe experiment was conducted using a targeted therapy capsule robot prototype with proposed three-dimensional spindle-shaped receiving coil. Under the wireless power-transfer system, the capsule robot could move along the tube steadily, and even make turns. The average speed of the capsule robot was 1.43 mm/s. The results show that stable and sufficient power could be supplied by the proposed receiving coil for the capsule robot in terms of misalignment in position and angle between transmitting and receiving coils.

## Figures and Tables

**Table 1:** Optimization parameters of the receiving coil.

Parameters	Value
N/groups	3
L/layers	3
T/turns	17

**Table 2:** Parameters of the transmitting coil.

Parameters	Value
Diameter/mm	400
Turns No.	20
Wire	Litz wire, 180 strands, 0.12 mm diameter

## Data Availability

Not applicable.
